# Mean Micronuclei Score in Fine-Needle Aspiration Cytology of Patients with Malignant Breast Lump in the Department of Pathology in a Tertiary Care Centre: A Descriptive Cross-sectional Study

**DOI:** 10.31729/jnma.7909

**Published:** 2022-12-31

**Authors:** Mona Dahal, Paricha Upadhyaya, Purbesh Adhikari, Niraj Regmi

**Affiliations:** 1 Department of Pathology, B.P. Koirala Institute of Health Sciences, Dharan, Sunsari, Nepal; 2 Department of Radiodiagnosis and Imaging, B.P. Koirala Institute of Health Sciences, Dharan, Sunsari, Nepal

**Keywords:** *biomarker*, *breast neoplasm*, *chromosomal instability*, *fine-needle aspiration*

## Abstract

**Introduction:**

Micronucleus is used as a biomarker of chromosomal instability, which is one of the hallmarks of neoplastic transformation. As micronuclei score increases with malignancy, it can be an effective and inexpensive adjunct to breast fine needle aspiration cytology, in diagnosing breast lumps, especially detecting grey lesions between benign and malignant tumours. The aim of this study is to find out the mean micronuclei score in fine-needle aspiration cytology of patients with malignant breast lumps in the Department of Pathology in a tertiary care centre.

**Methods:**

A descriptive cross-sectional study was conducted among patients with malignant breast lumps in the Department of Pathology in a tertiary care centre between 1 May 2020 to 31 May 2021 after receiving ethical approval from the Institutional Review Committee (Reference number: IRC/2139/021). The fine-needle aspiration cytology of breast lumps was diagnosed as per National Health services breast screening program guidelines. The mean micronuclei score was calculated. Convenience sampling was done and data were collected from the hospital records in the Department. Point estimate and 95% Confidence Interval were calculated.

**Results:**

Among 20 malignant breast aspirates, the mean micronuclei score was found to be 8.30±3.75 (3-19, 95% Confidence Interval).

**Conclusions:**

The mean micronuclei score in fine-needle aspiration cytology of malignant breast lumps was found to be similar when compared to similar studies conducted in similar settings.

## INTRODUCTION

Breast cancer confers a significant public health problem in developing countries. Various nuclear abnormalities like chromatin bridges, multipolar mitoses, micronuclei (MN) and nuclear buds, that play a crucial role in breast carcinogenesis have been recognized as a marker of chromosome instability, which can be identified on light microscopy.^1-4^

Fine needle aspiration cytology (FNAC) has been widely recognized as a minimally invasive and reliable diagnostic tool for diagnosing breast lesions. However, in various instances, cytological reports may be inconclusive or equivocal and can pertain to false negative or false positive results.^2,5^ As micronuclei score increases with malignancy, it can be an effective and inexpensive adjunct to breast FNAC, in diagnosing breast lumps, especially detecting grey lesions between benign and malignant tumours.

The aim of this study is to find out the mean micronuclei score in fine-needle aspiration cytology of malignant breast lumps in the Department of Pathology in a tertiary care centre.

## METHODS

A descriptive cross-sectional study was conducted among 20 malignant breast lumps in the Department of Pathology in a tertiary care centre between 1 May 2020 to 31 May 2021 after receiving ethical approval from the Institutional Review Committee of B.P. Koirala Institute of Health Sciences (Reference number: IRC/2139/021). All patients presenting with breast lumps who underwent FNAC and whose histopathological samples were available after FNAC procedure and the diagnosis of C5 (Malignant) were included in the study. The samples that had inadequate material for diagnosis and cases with non-epithelial lesion of breast were excluded from the study. Convenience sampling was done and the sample size was calculated using the formula,


$$\begin{array}{ll} \text{n} & ={\text{Z}}^{2}\times \frac{\sigma^{2}}{{\text{e}}^{\text{2}}}\\ & =1.96^{2}\times \frac{6.5^{2}}{3^{2}}\\ & =19 \end{array}$$

Where,

n = minimum required sample size
Z = 1.96 at 95% Confidence Interval (CI)
σ = standard deviation taken from published literature^6^
e = margin of error, 3%

The minimum required sample size was 19. However, a sample size of 20 was taken for the study. The relevant data and slides were collected from the archives of the department of pathology. The fine-needle aspiration cytology of breast lumps were diagnosed as per National Health Services Breast Screening Program (NHSBSP) guidelines: C1 (Inadequate), C2 (Benign), C3 (Atypia, probably benign), C4 (Suspicious of malignancy) and C5 (Malignant).^5^

For the identification and scoring of micronuclei, all the cytological smears of breast lump stained with Papanicolaou and Giemsa stain were evaluated under 100x objective lens (oil immersion). Micronuclei were identified and scored in epithelial cells using following criteria:^7^

1. Round to oval shape
2. 1/16 to 1/3 of the nuclear diameter
3. Not linked to main nucleus
4. Slightly darker/same staining intensity as main nucleus

Micronucleus were counted in first random 1000 epithelial cells on breast cytology smears under oil by authors blinded of the diagnosis on histopathology. All the epithelial cells with intact cell membrane were incorporated for identification of micronuclei.

Degenerated cells, cells with obscured or altered morphology and cells with clumped groups will be avoided. Epithelial cell with multiple micronuclei was given a score of 1. The malignant aspirates were further graded as Grade 1, 2 and 3 as per Robinson's Cytological Grading System.^8^

Data were entered and analysed using IBM SPSS Statistics version 22.0. Point estimate and 95% CI were calculated.

## RESULTS

Among 20 malignant breast aspirates, the mean micronuclei score was found to be 8.30±3.757 (319, 95% Confidence Interval). The histopathological diagnosis of all the 20 cases were malignant.

The mean MN score of the malignant aspirates with cytological grade 3 was 13.20±3.834 (10-19 at 95% Confidence Interval) (Table 1).

**Table 1:** Mean MN scores of the malignant aspirates (n= 20).

Cytological grade	n (%)	Mean±SD (Minimum-Maximum)
1	5 (25)	6.00±2.12 (3-8)
2	10 (50)	7.00±1.764 (5-11)
3	5 (25)	13.20±3.834 (10-19)

## DISCUSSION

Micronucleus is an eccentric chromosomal fragment or whole chromosome in the cytoplasm that fails to be incorporated in the main nucleus during cell division. As their appearance determines chromosomal breakage and loss, micronuclei have been used as a biomarker of chromosomal damage, genomic instability and cancer risk.^9,10^

The term micronucleus (MN) was first suggested by Boller and Schmidt in the 1970s. The utility of micronucleus assay to detect the genotoxic potential of mutagens after in vivo exposure of animals using bone marrow erythrocytes was shown by Heddle. Countryman and Heddle further recommended that peripheral blood lymphocytes could be used for micronucleus approach in 1976. Later in 1980, micronucleus assay was studied on exfoliated buccal mucosal cells to evaluate the genotoxic effects of betel nuts and quid. Since then, micronucleus scoring has been applied in evaluating genotoxicity of various carcinogens, pesticides; differentiating reactive versus metastasis in effusion cytology, screening of cancers of organs like cervix, breast, urothelial, oral cavity, lung, upper aerodigestive tract, colon; monitoring of treatment such as patients undergoing radiotherapy for nasopharyngeal carcinoma as well as in diseases related with genetic cause like diabetes mellitus and Down Syndrome.^7,11^

In our study, the mean micronuclei score was found to be 8.30±3.757 among 20 malignant breast aspirates. The MN score in our study was consistent with some studies done in India.. However, the MN score is slightly lower than the MN score depicted in few other studies done in India.^3,6,9,12^

The mean MN score increased with cytological grade with 6±2.12, 7±1.764 and 13.20±3.834 in Grade 1, Grade 2 and Grade 3 respectively. A greater degree of genetic instability in higher grades could confer the increase in MN score with increasing grades of malignant aspirates. The finding was concurrent with the results shown by various studies done in India. However, the MN score in grade 3 cases was very high (100±36.5) and slightly more i.e., 21.1±16.7 and 27.5±4.18 in a few studies done in India compared to our study. The MN score in our study is slightly lower than the MN score depicted in these studies as well.^3,4,9,10,12^

Breast carcinogenesis involves mutation in various genes associated with DNA repair and genetic stability, mainly BRCA1, BRCA2 and TP53. Moreover, BRCA1 and BRCA2 mutation is seen in 80-90% of single gene familial breast cancers and about 3-6% of all breast cancers. A study has shown an increase in MN score of exfoliated squamous cells of buccal mucosa of breast carcinoma patients compared to control. In addition, a study has depicted significant increase in MN score in buccal mucosa from controls to first degree female relatives of breast carcinoma patients and from first degree female relatives to breast carcinoma patients.^13-15^

We have performed MN scoring of malignant breast aspirates in Giemsa and Papanicolaou stained slides under light microscopy which are DNA non specific stains. The other DNA non specific stain for MN scoring includes May Grunwald Giemsa, Hematoxylin and Eosin and Crystal Violet. The DNA specific fluorescence stains have also been used for MN scoring. The DNA-specific fluorescence stains like DAPI (4',6-diamidino-2-phenylindole), Hoechst 33528, Acridine Orange and Propidium Iodide which require fluorescence microscope for the interpretation. Fluorescence stains are considered more sensitive for MN scoring due to the chances of misinterpretation of karyorrhexis, karyolysis, condensed chromatin as MN in DNA non specific stains. However, a study done in India has shown an increase in MN score from 9.70±6.5 in Giemsa-stained slides of malignant breast aspirates to 11.28±7.22 in Acridine Orange stained slides. However, few authors have suggested cautious interpretation of MN in Giemsa-stained slides due to their overestimation in presence of other nuclear anomalies. We have performed MN scoring in Papanicolaou and Giemsa-stained slides under light microscope considering the unavailability of fluorescence microscope in our resource setting and routine use of Papanicolaou and Giemsa stain in FNAC.^6,16,17^

## CONCLUSIONS

The mean micronuclei score in fine-needle aspiration cytology of malignant breast lumps was found to be similar when compared to similar studies conducted in similar settings.
